# High Cell-Free DNA Integrity Is Associated with Poor Breast Cancer Survival

**DOI:** 10.3390/cancers13184679

**Published:** 2021-09-18

**Authors:** Maria Lamminaho, Jouni Kujala, Hanna Peltonen, Maria Tengström, Veli-Matti Kosma, Arto Mannermaa

**Affiliations:** 1 Institute of Clinical Medicine, Pathology and Forensic Medicine, University of Eastern Finland, FI-70211 Kuopio, Finland; mariahm@student.uef.fi (M.L.); jouni.kujala@uef.fi (J.K.); hanna.peltonen@uef.fi (H.P.); veli-matti.kosma@uef.fi (V.-M.K.); 2 Cancer Center, Kuopio University Hospital, FI-70029 Kuopio, Finland; maria.tengstrom@kuh.fi; 3 Department of Clinical Pathology, Kuopio University Hospital, FI-70029 Kuopio, Finland; 4 Multidisciplinary Cancer Research Community (RC Cancer), University of Eastern Finland, FI-70211 Kuopio, Finland; 5 Biobank of Eastern Finland, Kuopio University Hospital, FI-70029 Kuopio, Finland

**Keywords:** liquid biopsy, biomarker, DNA fragmentation, diagnostics

## Abstract

**Simple Summary:**

A recent point of focus in breast cancer (BC) research has been the utilization of cell-free DNA and its concentration (cfDConc) and integrity (cfDI) as potential biomarkers. Though the association of cfDConc and BC survival is already recognized, studies on the prognostic value of cfDI have had contradictory results. The aim of this study was to investigate the prognostic potential of cfDConc and cfDI in Eastern Finnish BC cases with a non-metastatic disease. While the prognostic value of cfDConc remained non-significant in our analyses, high cfDI was an independent prognostic factor for poor overall survival (OS) and breast cancer-specific survival (BCSS). Inclusion of cfDI in the multivariate logistic regression model improved the predictive performance of the model, thus suggesting that the combined use of traditional tumor features and liquid biopsy could help to discriminate BC patients with poor OS and BCSS more accurately at the time of diagnosis.

**Abstract:**

Background: A recent point of focus in breast cancer (BC) research has been the utilization of cell-free DNA (cfDNA) and its concentration (cfDConc) and integrity (cfDI) as potential biomarkers. Though the association of cfDConc and poor survival is already recognized, studies on the prognostic value of cfDI have had contradictory results. Here, we provide further evidence to support the use of cfDI as a potential biomarker. Methods: We selected 204 Eastern Finnish BC cases with non-metastatic disease and isolated cfDNA from the serum collected at the time of diagnosis before any treatment was given. The cfDConc and cfDI were measured with a fluorometer and electrophoresis and analyzed with 25 years of survival data. Results: High cfDConc was not an independent prognostic factor in our analyses while high cfDI was found to be an independent prognostic factor for poor OS (*p* = 0.020, hazard ratio (HR) = 1.57, 95% confidence interval (CI) 1.07–2.29, Cox) and BCSS (*p* = 0.006, HR = 1.93, 95% CI 1.21–3.08)). Inclusion of cfDI in the multivariate logistic regression model improved the predictive performance. Conclusions: Our results show high cfDI is an independent prognostic factor for poor OS and BCSS and improves the predictive performance of logistic regression models, thus supporting its prognostic potential.

## 1. Introduction

Breast cancer (BC) is the most common cancer among women, with more than two million new cases diagnosed in 2018 [1]. The prognosis for non-metastasized BC is generally good and the 5-year survival in developed countries is currently ≥85% [2]. However, survival rates vary between different BC subtypes and approximately 20–30% of BC patients will eventually develop recurrent disease [3]. Earlier and more accurate identification of BC patients with a poor prognosis and tendency for recurrent disease is a key factor in reducing BC mortality.

A recent point of interest in BC research has been the utilization of cell-free DNA (cfDNA) for diagnostic purposes, as it could provide a non-invasive and easily repeated method of identifying patients with aggressive BC. It is well established that patients with advanced BC have higher cfDNA concentrations (cfDConc) than healthy controls or patients with benign tumors [4,5]. However, a high cfDConc has also been observed in other pathological conditions [6,7] which makes it relatively non-specific and highlights the need for more accurate biomarkers.

Various pathways are known to release cfDNA into circulation. In healthy individuals, cfDNA is mostly derived from apoptotic cells, in which endonuclease activity fragments the chromatin into nucleosomal units of approximately 180 bp and its multiples thereof. Longer fragments of >1000 bp in size are considered to originate especially from necrotic cells and other release mechanisms such as pyroptosis and phagocytosis [8,9]. The cfDNA integrity (cfDI) describes the ratio of large and small cfDNA fragments. It is well established that BC patients generally have higher cfDI than healthy controls or patients with benign disease [10,11,12,13,14], suggesting that the role of long fragment producing release mechanisms plays a central role in BC.

As tumor necrosis is often associated with a poor prognosis [15], high cfDI may be associated with a poor prognosis and act as a more specific biomarker of aggressive BC. Here, we isolated cfDNA from serum samples from patients with invasive local disease and measured the cfDConc and cfDI to compare their prognostic potential in estrogen receptor (ER) positive and negative BC.

## 2. Materials and Methods

### 2.1. Patients, Sample Material and Clinical Data

This study included a set of 207 Eastern Finnish BC patients. Their serum samples were collected at the time of diagnosis before receiving any kind of treatment. All patients were diagnosed with invasive local disease. Patient characteristics are presented in Table 1. Sample material and clinical data was obtained from KBCP, a prospective population-based case-control study conducted in 1990–1995 in Eastern Finland, and follow-up is still ongoing. This research project was advocated by the Research Ethics Committee of the University of Eastern Finland and Kuopio University Hospital. All participants have given their knowledge based written consent to participation.

### 2.2. Isolation of Cell-Free DNA

Serum samples were thawed on ice and 1 mL of serum was used for isolation. Each sample was centrifuged at 2000× *g* for 10 min at room temperature to remove possible remnants of white cell debris. The cfDNA was isolated from samples by using the QIAamp Circulation Nucleic Acid Kit (Cat. No. 55114, Qiagen, Hildern, Germany) according to manufacturer’s protocol. Isolated cfDNA was eluted in 50 L of AVE buffer and stored at −20 °C.

### 2.3. Concentration and Fragmentation Measurements

The concentration of isolated cfDNA samples was measured using Qubit 2.0 (Thermo Fischer Scientific, Waltham, MA, USA) fluorometer. A TapeStation 4200 (Agilent Technologies, Waltman, MA, USA) electrophoresis system with a ScreenTape D5000 System was used according to manufacturer’s protocol to evaluate the cfDI. According to Jahr et al. [16], fragments of 180 bp and multiples thereof were considered to be apoptotic fragments, and fragments in the range of 3000–10,000 bp were considered to be necrotic fragments. Applying this information to our dataset, we decided to interpret the fragments ≤1000 bp as apoptotic and larger fragments as necrotic fragments (Supplementary Figure S1). An integrity score was calculated by dividing the peak area of necrotic fragments by the peak area of apoptotic fragments.

### 2.4. Statistical Analysis

Statistical analyses were carried out in SPSS Statistics 25 software (IBM, Armonk, NY, USA). Concentrations smaller than the Qubit 2.0 threshold of 0.1 pg/µL were imputed as 0.00 ng/µL. Survival analysis was performed using the Kaplan–Meier estimate and Cox proportional hazards model with the following covariates: age at the time of diagnosis, tumor grade and stage, nodal status, ER status, progesterone receptor (PR) status, human epidermal growth factor receptor 2 (HER2) status, radiotherapy, chemotherapy, and hormonal therapy. The OS and BCSS were calculated as the time from the date of diagnosis to the date of last follow-up or death. Cause of death was coded either as caused by BC or not caused by BC. RFS was calculated as the time of first local or distant recurrence or new BC.

The prognostic potential of cfDConc and cfDI was further analyzed in univariate and multivariate logistic regression analysis. The variables age at the time of diagnosis, tumor grade and stage, nodal status, ER status, PR status, HER2 status, cfDConc, and cfDI were used to build the multivariate logistic regression models and the diagnostic performance of these models was estimated by a receiver operating characteristic (ROC) analysis and k-fold cross-validation score (Supplementary Figure S2). Bootstrapping with replacement was used to calculate area under curve (AUC) metric and its 95% confidence interval. A pairwise comparison of ROC curves was performed using DeLong’s algorithm [17].

The association between two variables was estimated by Pearson’s chi-squared test. Variable distributions between two groups were evaluated by Mann–Whitney’s U test. A *p*-value ≤ 0.05 (two-sided) was considered statistically significant in all analyses.

## 3. Results

### 3.1. Patient Cohort

All patients were female with a median age of 55 years; 138 patients (67.6%) were diagnosed with ER+ BC and 66 patients (32.4%) with ER− BC. The most common histological subtypes were ductal carcinoma (72.5%) and lobular carcinoma (14.2%). All patients had undergone breast surgery after primary diagnosis and sampling. Most of the patients had also received radiotherapy (78.9%) but only a minority of patients had received chemotherapy (21.1%) or hormonal therapy (25.0%).

**Figure 1:**
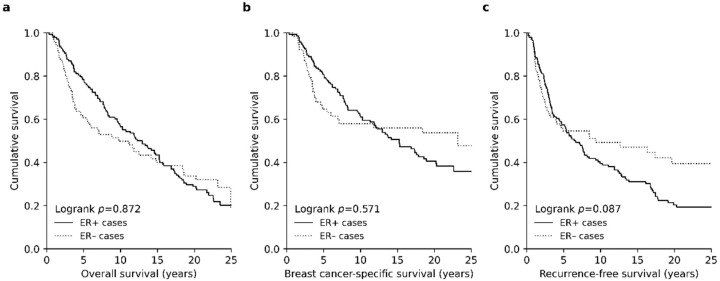
Survival functions in the ER+ and ER− patients. (**a**) ER− BC was associated with poor short-term overall survival and (**b**) breast cancer-specific survival especially during the first five years of follow-up compared to ER+ BC. (**c**) The difference in survival was lifted off over time, mainly due to different recurrence rates on long-term follow-up.

At the time of the latest follow-up in October 2016, 52.9% of patients had died of BC and 21.1% of other cause. Of the 26.0% of patients who were alive, approximately two-fifths (37.7%) had been diagnosed with recurrent disease. The short-term survival of ER- patients was generally worse than patients with ER+ disease; the five-year overall survival (OS) and breast cancer-specific survival (BCSS) rates were 78.3% and 80.6% for ER+ cohort while corresponding survival rates for ER− BC were 60.6% and 64.5% (Figure 1a,b). The risk of recurrent disease was highest during the first five years of follow-up, after which the risk of recurrence in ER− patients was strongly reduced (Figure 1c).

### 3.2. High cfDConc Is Not Associated with Poor BC Survival

**Figure 2:**
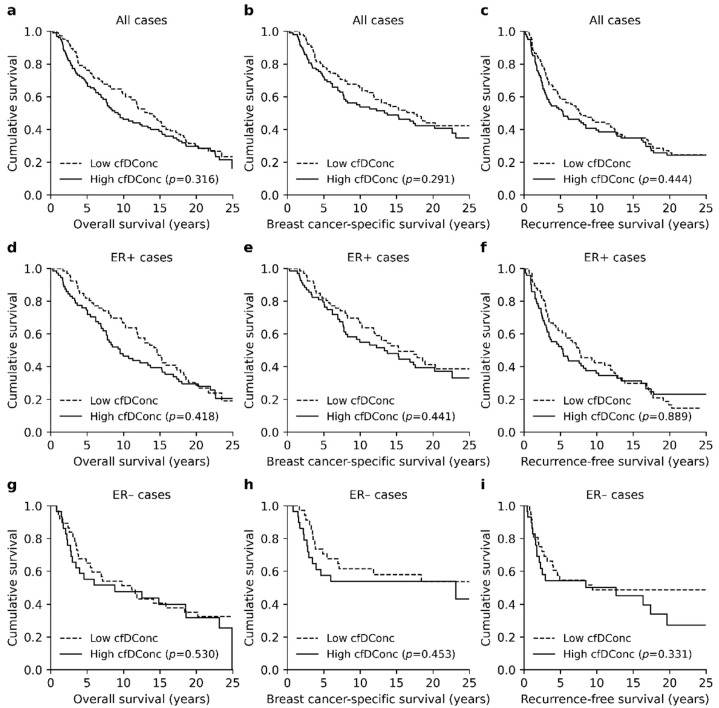
Kaplan–Meier plots representing the association between cfDConc and BC survival. (**a**–**c**) Patients with high cfDConc had generally worse survival, although the only significant difference in survival was observed for OS at the 10-year follow-up (*p* = 0.022). (**d**–**f**) A similar survival trend was observed in the ER+ patients with a significant association with OS at the 10-year follow-up (*p* = 0.017). (**g**–**i**) The association with poor survival was less prominent in the ER− patient cohort.

The median cfDConc was 0.56 ng/µL with a range of 0.20–2.82 ng/µL before imputation. When all patients were divided into two groups, according to the median cfDConc (high and low cfDConc), patients with high cfDConc had generally worse survival (Figure 2a–c). However, the only significant difference in survival was observed for OS at the 10-year follow-up (*p* = 0.022, Logrank, Table S1).

No difference in cfDConc was observed between ER+ and ER− patients (*p* = 0.208, Mann–Whitney U). ER+ patients with a high cfDConc had generally worse survival (Figure 2d–f), which was most prominent for the OS at the 10-year follow-up (*p* = 0.017, Logrank). No notable differences in survival were observed in the ER− patient group (Figure 2g–i).

Multivariate survival analysis did not recognize high cfDConc as an independent prognostic factor (Figure S3) and the difference in patient survival was mainly explained by tumor stage, lymph node status and age at the time of diagnosis. Similarly, cfDConc was strongly associated with tumor stage (*p* = 0.005, Pearson’s chi-square) and lymph node status (*p* = 0.009).

### 3.3. High Cfdi Is An Independent Prognostic Factor for Poor OS and BCSS

**Figure 3:**
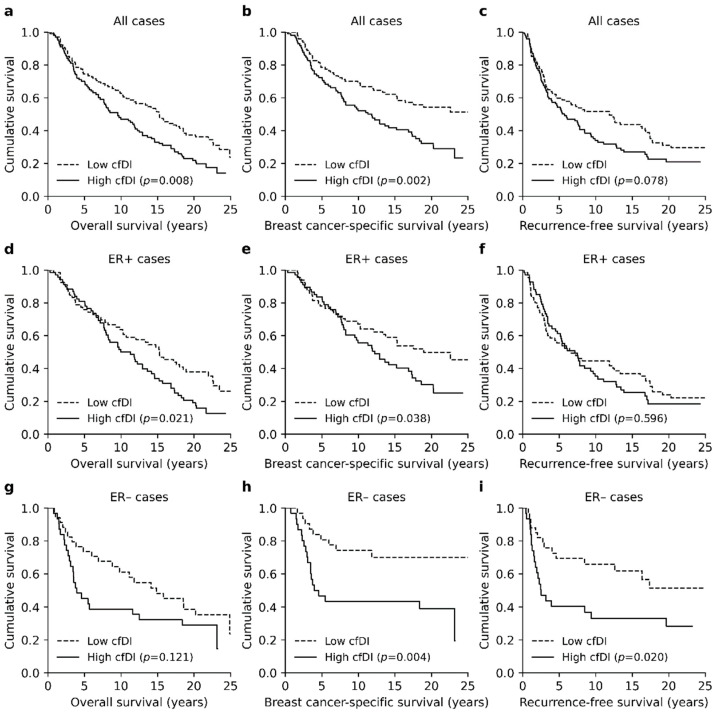
Kaplan–Meier plots representing the association between cfDI and survival. (**a**) High cfDI was associated with poor OS (*p* = 0.002) and (**b**) BCSS (*p* = 0.008). (**c**) The association with RFS was less prominent and only visible at the 10- and 15-year follow-ups (*p* = 0.045 and *p* = 0.037). (**d**,**e**) In ER+ patients, high cfDI was associated with poor OS and BCSS, but (**f**) association with RFS was not observed. (**g**) In ER− patients, high cfDI was associated with poor OS and (**h**) poor BCSS at the 5- and 10-year follow-ups (*p* = 0.024 and *p* = 0.045). (**i**) Association between high cfDI and poor RFS was the most prominent in ER− patients. All *p*-values were obtained by the Logrank test.

Calculated cfDI scores ranged from 0.05 to 3.37 with a median cfDI of 0.42. When all patients were divided according to the median cfDI score (high and low cfDI), high cfDI was associated with poor OS and BCSS (*p* = 0.008 and *p* = 0.002, Logrank, Figure 3a,b) in the univariate survival analysis. Association with poor recurrence-free survival (RFS) was most prominent at the 10- and 15-year follow-ups (*p* = 0.045 and *p* = 0.037, Logrank, Figure 3c).

Patients with ER+ BC had slightly higher median cfDI than ER− patients (0.64 ± 0.04 vs. 0.52 ± 0.07, *p* = 0.011, Mann–Whitney U). Further analysis did not find any explanatory factor for the observed difference in cfDI. High cfDI was associated with generally worse survival for both in the ER+ and ER− patients. In the ER+ group, high cfDI was associated with both poor OS and poor BCSS (*p* = 0.021 and *p* = 0.038, Logrank, Figure 3d,e), but a notable association with RFS was not observed (*p* = 0.596, Figure 3f). In the ER− group, patients with a high cfDI had worse OS (*p* = 0.004, Figure 3g) and BCSS, especially at the 10-year follow-up (*p* = 0.045, Figure 3h). Moreover, high cfDI was associated with poor RFS in ER− patients (*p* = 0.020, Figure 3i).

**Figure 4:**
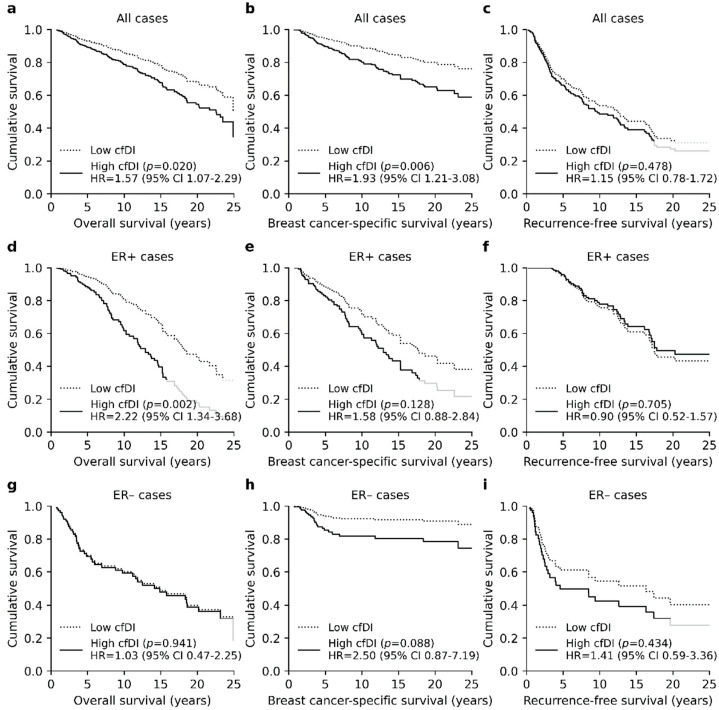
Cox regression analysis plots representing the association between high cfDI and BC survival. (**a**) High cfDI was an independent prognostic factor for poor OS (*p* = 0.006) and (**b**) BCSS (*p* = 0.020) but not for the (**c**) poor RFS (*p* = 0.478). (**d**–**f**) Similar results were observed in ER+ patients although the only significant difference in survival was observed for OS. (**g**–**i**) In the ER− patients high cfDI was associated with poor BCSS but was not significant, whereas associations with OS and RFS remained less prominent. Grey lines represent the survival function of high cfDI group in areas where the plot overlaps with the legend.

Multivariate survival analysis indicated that cfDI is an independent prognostic factor for poor OS (*p* = 0.020, hazard ratio (HR) = 1.57, 95% confidence interval (CI) 1.07–2.29, Figure 4a) and BCSS (*p* = 0.006, HR = 1.93, 95% CI 1.21–3.08, Figure 4b) when dividing patients according to the median cfDI. The association with RFS was not prominent (*p* = 0.478, Figure 4c). In the ER+ patient group, high cfDI was an independent prognostic factor for poor OS (*p* = 0.002, HR = 2.22, 95% CI 1.34–3.68, Figure 4d), but the associations with BCSS and RFS were less prominent (*p* = 0.128 and *p* = 0.705, Figure 4e,f). In the ER− patient group, high cfDI was associated with poor BCSS (*p* = 0.088, Figure 4g) but remained non-significant in our analysis. Associations with OS and RFS were not notable (*p* = 0.941 and *p* = 0.434, Figure 4h,i). In general, the survival of the ER− patients was explained mainly by the tumor stage and positive lymph node status.

### 3.4. The cfDI Improves the Prediction Accuracy of Logistic Regression Models

Multivariate logistic regression model with key tumor features and patient age at the time of diagnosis was considered as a reference model to which other logistic regression models were compared to estimate how cfDConc and cfDI can improve the prediction of clinical outcomes. Univariate logistic regression models with cfDConc as an only predictor did not significantly differ from the use of a random classifier; the measured area under curves (AUCs) for OS, BCSS, and RFS were 0.510 (95% CI 0.425–0.591), 0.453 (95% CI 0.391–0.537), and 0.440 (95% CI 0.361–0.517), respectively (Table S2, Figure S4). Inclusion of cfDConc did not significantly improve the performance of the multivariate logistic regression model (Table S3).

**Figure 5:**
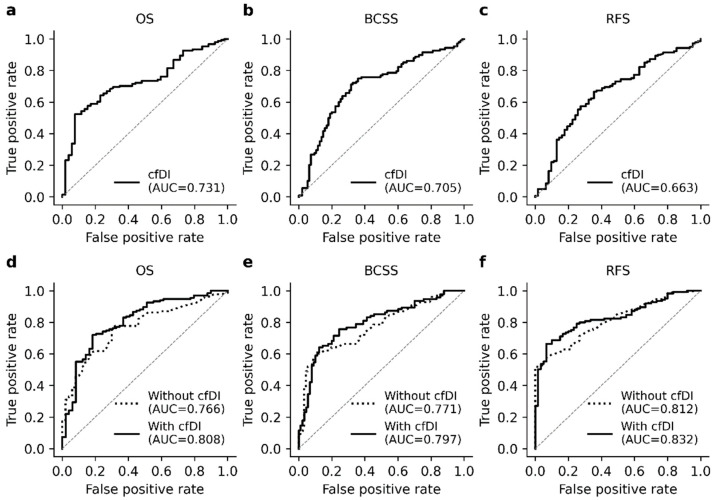
Performance of the univariate and multivariate logistic regression models with cfDI. The top row (**a**–**c**) represents the ROC curves derived from the univariate logistic analyses with cfDI as a predictor. The ROC curves illustrate that cfDI predicts OS and BCSS with an acceptable accuracy but is less accurate biomarker for BC recurrence. The bottom row (**d**–**f**) represents the performance of the multivariate logistic regression model with and without cfDI and illustrates how including the cfDI in the model improves the general model performance.

Univariate logistic regression with cfDI predicted the clinical outcomes of OS (AUC = 0.731, 95% CI 0.683–0.780), BCSS (AUC = 0.705, 95% CI 0.659–0.751) and RFS (AUC = 0.663, 95% CI 0.612–0.719) with an acceptable accuracy (Figure 5a–c). Multivariate logistic regression with key tumor features and patient age outperformed the univariate analysis (Figure 5d–f) in all cases, and inclusion of cfDI in the multivariate analysis further improved the general performance of the models. The AUCs obtained for multivariate logistic regression models with key tumor features and cfDI were 0.808 (95% CI 0.760–0.859), 0.797 (95% CI 0.757–0.841), and 0.832 (95% CI 0.796–0.871) for OS, BCSS, and RFS, respectively. The improvement was significant only in models predicting OS and BCSS (*p* = 0.057 and *p* < 0.001, DeLong); the model predicting RFS did not significantly benefit from the inclusion of cfDI (*p* = 0.977).

## 4. Discussion

The association between high cfDI and cancer indicates that high cfDI could also be associated with poor survival, but the few studies that have focused on the subject have reported controversial results. Iqbal et al. reported that high cfDI is associated with poor RFS in newly diagnosed BC patients [18], whereas Cheng et al. and Madhavan et al. reported opposite results and connected high cfDI to better OS and RFS in patients with metastatic BC [19,20]. Furthermore, Medhavan et al. did not support the prognostic value of cfDI in patients with non-metastatic disease. Our results provide further evidence by identifying high cfDI as an independent prognostic factor for both poor OS and BCSS at the time of diagnosis and before any treatment is given to patients.

The noted studies have important differences in terms of study design, patient cohort used, and especially timing of sample collection. Our methodology does differ from previous studies as we decided to use automated gel electrophoresis instead of Alu-based real-time PCR in order to allow the visual inspection of the fragmentation and evaluate the whole spectrum of cfDNA fragments over the 100–5000 bp range. Moreover, we chose to use a higher and more biologically relevant threshold for the detection of necrotic fragments. Though differences in the methodology and patient cohorts may partly explain the controversial results, it is obvious that more research and systematic reviews on the topic are needed to validate the prognostic potential of cfDI.

Although our results support, to some extent, the prognostic potential of cfDConc, we did not recognize cfDConc as an independent prognostic factor due to its strong association with tumor stage and lymph node status. This also explains why inclusion of cfDConc in the logistic regression model did not provide notable improvement in the prediction accuracy, as survival was mainly explained by tumor stage and lymph node status. The inclusion of cfDI, in turn, improved the prediction accuracy due to cfDI not being strongly associated with traditional tumor features but being strongly associated with the poor survival. Cross-validation confirmed that models with cfDConc as a predictor suffered from varying accuracy while models with cfDI and traditional tumor features provided more robust performance.

Particularly interesting is the association of cfDI and survival in ER+ patients. This group of patients had a uniform OS and BCSS pattern during the first five years of survival, after which the patients with a high cfDI had significantly worse survival than patients with low cfDI. This observation highlights the prognostic potential of cfDI but also raises questions about the exact mechanism underlying the observed difference. Why did these cases have such poor survival, and why did the difference in survival manifest so late? This group of patients did not differ from the low cfDI group in terms of tumor features or received treatments which suggests that liquid biopsy might reflect something that traditional tumor biopsies miss.

The basic mechanism underlying the prognostic potential of cfDI remains open for discussion. Our hypothesis assumes that increased cfDI results from tumor necrosis and is therefore associated with poor BC survival. While our results support the association between high cfDI and poor survival we cannot conclusively connect this finding with the tumor necrosis due to available sample material. At the time of sampling, necrotic tumor regions were consciously avoided in sample preprocessing and remaining samples did not perfectly reflect the presence of tumor necrosis. Although the role of necrotic tumor cells as a major source of long cfDNA fragments is more or less accepted [21,22], the association between the high cfDI and tumor necrosis still needs more supportive evidence.

The sample material used has both strengths and limitations regarding its use. Long-term storage and use of serum samples is associated with pre-analytical problems, such as hematopoietic lysis and considerable gDNA contamination [23], which is why careful quality control was applied. Moreover, the treatment of BC has developed substantially after the Kuopio Breast Cancer Project (KBCP), and how well our results can be generalized to modern clinical practice is open for debate. On the other hand, the exceptionally long follow-up of 25 years provides a unique prospective perspective regarding the survival of Finnish BC patients and allowed us to perform both short- and long-term survival analyses that would have not been possible with a standard 5-year follow-up.

## 5. Conclusions

Association of cfDI and BC survival is poorly studied and few studies have focused on the topic with notable differences in terms of study design and the obtained results. Our results provide further evidence of the association between cfDI and BC survival and recognize cfDI as an independent prognostic factor for poor BCSS, especially in ER+ BC. Together with the traditional tumor features, a non-invasive and easily repeated liquid biopsy could help discriminate BC patients with poor OS and BCSS more accurately at the time of diagnosis. Further studies and systematic reviews on the topic are well warranted.

## Figures and Tables

**Table 1:** Patient characteristics.

Variable	Grouping	Total	ER+	ER−
Age at diagnosis	≤39 years	25 (12.3%)	15 (10.9%)	10 (15.2%)
	40–49 years	47 (23.0%)	30 (21.7%)	17 (25.8%)
	50–59 years	56 (27.5%)	36 (26.1%)	20 (30.3%)
	60–69 years	37 (18.1%)	27 (19.6%)	10 (15.2%)
	≥70 years	39 (19.1%)	30 (21.7%)	9 (13.6%)
Tumor stage	I	81 (39.7%)	59 (42.8%)	22 (33.3%)
	II	98 (48.0%)	66 (47.8%)	32 (48.5%)
	II	25 (12.3%)	12 (9.4%)	12 (18.2%)
Tumor grade	I	36 (17.6%)	34 (24.6%)	2 (3.0%)
	II	97 (47.5%)	78 (56.5%)	19 (28.8%)
	II	70 (43.3%)	25 (18.1%)	45 (68.2%)
Nodal status	N0	115 (56.4%)	80 (58.0%)	35 (53.0%)
	N1	79 (38.7%)	53 (38.4%)	26 (39.4%)
	N2	10 (4.9%)	5 (3.6%)	5 (7.6%)
PR status	Negative	95 (46.6%)	34 (24.6%)	61 (92.4%)
	Positive	109 (53.4%)	104 (75.4%)	5 (7.6%)
HER2 status	Negative	173 (84.8%)	122 (88.4%)	51 (77.3%)
	Positive	17 (8.3%)	4 (2.9%)	13 (19.7%)
	Missing	14 (6.9%)	12 (8.7%)	2 (3.0%)
Chemotherapy	Yes	43 (21.1%)	21 (15.2%)	22 (33.3%)
	No	161 (78.9%)	117 (84.8%)	44 (66.7%)
Hormonal therapy	Yes	51 (25.0%)	40 (29.0%)	11 (16.7%)
	No	153 (75.0%)	98 (71.0%)	55 (83.3%)
Radiotherapy	Yes	129 (63.2%)	86 (62.3%)	43 (65.2%)
	No	75 (36.8%)	52 (37.7%)	23 (34.8%)

## Data Availability

The datasets used and analyzed during the study are available from the corresponding author on reasonable request.

## Supplementary Materials

The following are available online at https://www.mdpi.com/article/10.3390/cancers13184679/s1, Table S1: Logrank results at different follow-up checkpoints, Table S2: Receiver operator characteristic (ROC) curve metrics, Table S3: Pairwise ROC curve comparisons, Figure S1: Peak area analysis of cfDNA integrity, Figure S2: Cross-validation of logistic regression models, Figure S3: Cox regression plots of high cfDConc and BC survival, Figure S4: Performance of univariate and multivariate logistic regression models.

## References

[1] Sung H., Ferlay J., Siegel R.L., Laversanne M., Soerjomataram I., Jemal A., Bray F. (2021). Global Cancer Statistics 2020: Globocan Estimates of Incidence and Mortality Worldwide for 36 Cancers in 185 Countries. CA Cancer J. Clin..

[2] Allemani C., Matsuda T., Di Carlo V., Harewood R., Matz M., Nikšić M., Bonaventure A., Valkov M., Johnson C.J., Estève J. (2018). Global surveillance of trends in cancer survival 2000–2014 (CONCORD-3): Analysis of individual records for 37,513,025 patients diagnosed with one of 18 cancers from 322 population-based registries in 71 countries. Lancet.

[3] Riggio A.I., Varley K.E., Welm A.L. (2021). The lingering mysteries of metastatic recurrence in breast cancer. Br. J. Cancer.

[4] Tan G., Chu C., Gui X., Li J., Chen Q. (2018). The prognostic value of circulating cell-free DNA in breast cancer. Medicine.

[5] Yu D., Tong Y., Guo X., Feng L., Jiang Z., Ying S., Jia J., Fang Y., Yu M., Xia H. (2019). Diagnostic Value of Concentration of Circulating Cell-Free DNA in Breast Cancer: A Meta-Analysis. Front. Oncol..

[6] Jylhävä J., Lehtimäki T., Jula A., Moilanen L., Kesäniemi Y.A., Nieminen M.S., Kähönen M., Hurme M. (2014). Circulating cell-free DNA is associated with cardiometabolic risk factors: The Health 2000 Survey. Atherosclerosis.

[7] Duvvuri B., Lood C. (2019). Cell-Free DNA as a Biomarker in Autoimmune Rheumatic Diseases. Front. Immunol..

[8] Wang W., Kong P., Ma G., Li L., Zhu J., Xia T., Xie H., Zhou W., Wang S. (2017). Characterization of the release and biological significance of cell-free DNA from breast cancer cell lines. Oncotarget.

[9] Grabuschnig S., Bronkhorst A.J., Holdenrieder S., Rodriguez I.R., Schliep K.P., Schwendenwein D., Ungerer V., Sensen C.W. (2020). Putative Origins of Cell-Free DNA in Humans: A Review of Active and Passive Nucleic Acid Release Mechanisms. Int. J. Mol. Sci..

[10] Wang B., Huang H.-Y., Chen Y.-C., Bristow R.E., Kassauei K., Cheng C.-C., Roden R., Sokoll L.J., Chan D.W., Shih I.-M. (2003). Increased plasma DNA integrity in cancer patients. Cancer Res..

[11] Umetani N., Giuliano A.E., Hiramatsu S.H., Amersi F., Nakagawa T., Martino S., Hoon D.S. (2006). Prediction of Breast Tumor Progression by Integrity of Free Circulating DNA in Serum. J. Clin. Oncol..

[12] Deligezer U., Eralp Y., Akisik E.E., Akisik E.Z., Saip P., Topuz E., Dalay N. (2008). Size distribution of circulating cell-free DNA in sera of breast cancer patients in the course of adjuvant chemotherapy. Clin. Chem. Lab. Med..

[13] Kamel A.M., Teama S., Fawzy A., El Deftar M. (2015). Plasma DNA integrity index as a potential molecular diagnostic marker for breast cancer. Tumor Biol..

[14] Miao Y., Fan Y., Zhang L., Ma T., Li R. (2019). Clinical value of plasma cfDNA concentration and integrity in breast cancer patients. Cell. Mol. Biol..

[15] Maiorano E., Regan M.M., Viale G., Mastropasqua M.G., Colleoni M., Castiglione-Gertsch M., Price K.N., Gelber R.D., Goldhirsch A., Coates A.S. (2010). Prognostic and predictive impact of central necrosis and fibrosis in early breast cancer: Results from two International Breast Cancer Study Group randomized trials of chemoendocrine adjuvant therapy. Breast Cancer Res. Treat..

[16] Jahr S., Hentze H., Englisch S., Hardt D., Fackelmayer F.O., Hesch R.D., Knippers R. (2001). DNA fragments in the blood plasma of cancer patients: Quantitations and evidence for their origin from apoptotic and necrotic cells. Cancer Res..

[17] Sun X., Xu W. (2014). Fast Implementation of DeLong’s Algorithm for Comparing the Areas under Correlated Receiver Operating Characteristic Curves. IEEE Signal Process. Lett..

[18] Iqbal S., Vishnubhatla S., Raina V., Sharma S., Gogia A., Deo S.S.V., Mathur S.R., Shukla N.K. (2015). Circulating cell-free DNA and its integrity as a prognostic marker for breast cancer. Springerplus.

[19] Madhavan D., Wallwiener M., Bents K., Zucknick M., Nees J., Schott S., Cuk K., Riethdorf S., Trumpp A., Pantel K. (2014). Plasma DNA integrity as a biomarker for primary and metastatic breast cancer and potential marker for early diagnosis. Breast Cancer Res. Treat..

[20] Cheng J., Holland-Letz T., Wallwiener M., Surowy H., Cuk K., Schott S., Trumpp A., Pantel K., Sohn C., Schneeweiss A. (2018). Circulating free DNA integrity and concentration as independent prognostic markers in metastatic breast cancer. Breast Cancer Res. Treat..

[21] Kustanovich A., Schwartz R., Peretz T., Grinshpun A. (2019). Life and death of circulating cell-free DNA. Cancer Biol. Ther..

[22] Rostami A., Lambie M., Yu C.W., Stambolic V., Waldron J.N., Bratman S.V. (2020). Senescence, Necrosis, and Apoptosis Govern Circulating Cell-free DNA Release Kinetics. Cell Rep..

[23] Chan K.A., Yeung S.-W., Lui W.-B., Rainer T., Lo Y.D. (2005). Effects of Preanalytical Factors on the Molecular Size of Cell-Free DNA in Blood. Clin. Chem..

