# Supplementary material for: High Cell-Free DNA Integrity Is Associated with Poor Breast Cancer Survival
Source: Cancers (Basel). 2021 Sep 18;13(18):4679. doi: 10.3390/cancers13184679 (PMC8467852; doi:10.3390/cancers13184679)
Supplement: Supplementary file 1 [file cancers-13-04679-s001.zip › cancers-1368404-supplementary.pdf]

cancers MDPI

Supplementary Materials: High Cell-Free DNA Integrity Is Associated with Poor Breast Cancer Survival

Maria Lamminaho, Jouni Kujala, Hanna Peltonen, Maria Tengström, Veli-Matti Kosma and Arto Mannermaa

This file contains supplementary material for the manuscript High cell-free DNA concentration and integrity are associated with a poor breast cancer survival by Lamminaho M., Kujala J., Peltonen H., Tengström M., Kosma VM., and Mannermaa A. (2021).

List of tables

Supplementary Table S1. Logrank test results at different follow-up checkpoints. S1

Supplementary Table S2. Receiver operator characteristic (ROC) curve metrics S2

Supplementary Table S3. Pairwise ROC curve comparisons..S3

List of figures

Supplementary Figure S1. Peak area analysis of cfDNA integrity...S1

Supplementary Figure S2. Cross-validation of logistic regression models..S2

Supplementary Figure S3. Cox regression analysis plots of high cfDConc and BC survival.............................S3

Supplementary Figure S4. Performance of univariate and multivariate logistic regression models. S4

Supplementary Table S1. Logrank test results at different follow-up checkpoints for patients with high vs. low cfDConc and cfDI according to median values.

		cfDConc			cfDI		
**Checkpoint**	**Function**	**All**	**ER+**	**ER–**	**All**	**ER+**	**ER–**
5 years	OS	2.041 (0.153)	1.453 (0.228)	1.694 (0.193)	0.585 (0.444)	0.436 (0.509)	5.085 (0.024)
	BCSS	1.453 (0.228)	0.230 (0.632)	1.673 (0.196)	1.189 (0.275)	0.494 (0.482)	8.055 (0.005)
	RFS	1.694 (0.193)	1.706 (0.192)	0.065 (0.798)	0.570 (0.450)	0.667 (0.414)	5.873 (0.015)
10 years	OS	5.259 (0.022)	5.724 (0.017)	0.263 (0.608)	4.373 (0.037)	1.721 (0.190)	4.030 (0.045)
	BCSS	3.184 (0.074)	1.987 (0.159)	0.896 (0.344)	5.265 (0.022)	1.230 (0.267)	7.098 (0.008)
	RFS	1.291 (0.256)	1.210 (0.271)	0.024 (0.877)	4.025 (0.045)	0.212 (0.645)	7.001 (0.008)
15 years	OS	2.502 (0.114)	2.301 (0.129)	0.317 (0.573)	6.008 (0.014)	3.491 (0.062)	2.616 (0.106)
	BCSS	1.861 (0.172)	0.904 (0.342)	0.968 (0.325)	8.490 (0.004)	3.051 (0.081)	7.030 (0.008)
	RFS	1.527 (0.468)	0.317 (0.573)	0.124 (0.725)	4.363 (0.037)	0.544 (0.461)	6.245 (0.012)
20 years	OS	0.836 (0.361)	0.714 (0.398)	0.170 (0.680)	6.279 (0.012)	4.666 (0.031)	2.137 (0.144)
	BCSS	0.796 (0.372)	0.492 (0.483)	0.351 (0.553)	8.281 (0.004)	3.502 (0.061)	6.992 (0.008)
	RFS	0.716 (0.397)	0.066 (0.797)	0.946 (0.331)	3.367 (0.067)	0.364 (0.546)	5.393 (0.020)
25 years	OS	1.007 (0.316)	0.657 (0.418)	0.795 (0.372)	7.109 (0.008)	5.349 (0.021)	2.400 (0.121)
	BCSS	1.114 (0.291)	0.593 (0.441)	0.394 (0.530)	10.038 (0.002)	4.304 (0.038)	8.472 (0.004)
	RFS	0.585 (0.444)	0.019 (0.889)	0.946 (0.331)	3.110 (0.078)	0.281 (0.596)	5.393 (0.020)

Results are provided as Logrank test statistic (level of significance). Abbreviations: cfDConc, cell-free DNA concentration; cfDI, cell-free DNA integrity score; OS, overall survival; BCSS, breast cancer-specific survival; RFS, recurrence-free survival.

Supplementary Table S2. Receiver operator characteristic (ROC) curve metrics. Area under the curve (AUC) metric is show with 95% confidence interval for each regression model. Statistic significance represents the comparison between indicated regression model and random classifier.

Included variables	Function	AUC (95% CI)	Sig.
cfDConc	OS	0.510 (0.425 - 0.591)	0.114
	BCSS	0.453 (0.391 - 0.537)	0.999
	RFS	0.440 (0.361 - 0.517)	1.000
cfDConc + tumor features	OS	0.787 (0.736 - 0.849)	<0.001
	BCSS	0.774 (0.714 - 0.835)	<0.001
	RFS	0.774 (0.722 - 0.824)	<0.001
cfDI	OS	0.733 (0.698 - 0.773)	<0.001
	BCSS	0.704 (0.661 - 0.758)	<0.001
	RFS	0.662 (0.610 - 0.702)	<0.001
cfDI + tumor features	OS	0.808 (0.764 - 0.851)	<0.001
	BCSS	0.799 (0.763 - 0.844)	<0.001
	RFS	0.829 (0.792 - 0.871)	<0.001

Abbreviations: cfDConc, cell-free DNA concentration; cfDI, cell-free DNA integrity score; OS, overall survival; BCSS, breast cancer-specific survival; RFS, recurrence-free survival.

Supplementary Table S3. Pairwise ROC curve comparisons. Area under the curve (AUC) metrics of the logistic regression models were compared with DeLong's algorithm.

Survival function[1]	Model 1	Model 2	AUC difference (95% CI)[2]	Z[3]	Sig
OS	cfDConc	tumor features	0.261 (0.253 – 0-270)	1.610	0.010
	cfDConc	cfDConc + tumor features	0.280 (0.271 – 0.289)	2.375	0.018
	cfDI	tumor features	0.037 (0.030 – 0.044)	1.577	0.115
	cfDI	cfDI + tumor features	0.073 (0.067 – 0.080)	2.913	0.004
BCSS	cfDConc	tumor features	0.316 (0.308 - 0.324)	3.018	0.003
	cfDConc	cfDConc + tumor features	0.324 (0.315 - 0.333)	3.403	<0.001
	cfDI	tumor features	0.066 (0.060 - 0.073)	4.372	<0.001
	cfDI	cfDI + tumor features	0.095 (0.089 - 0.102)	3.334	<0.001
RFS	cfDConc	tumor features	0.381 (0.372 - 0.390)	1.414	0.157
	cfDConc	cfDConc + tumor features	0.339 (0.329 - 0.349)	1.445	0.148
	cfDI	tumor features	0.149 (0.142 - 0.156)	3.992	<0.001
	cfDI	cfDI + tumor features	0.164 (0.158 - 0.171)	3.901	<0.001

[1]Abbreviations: OS, overall survival; BCSS, breast cancer-specific survival; RFS, recurrence-free survival; cfDConc, cell-free DNA concentration; cfDI, cell-free DNA integrity score. [2]AUC difference with 95% confidence interval. [3]Z-test value.

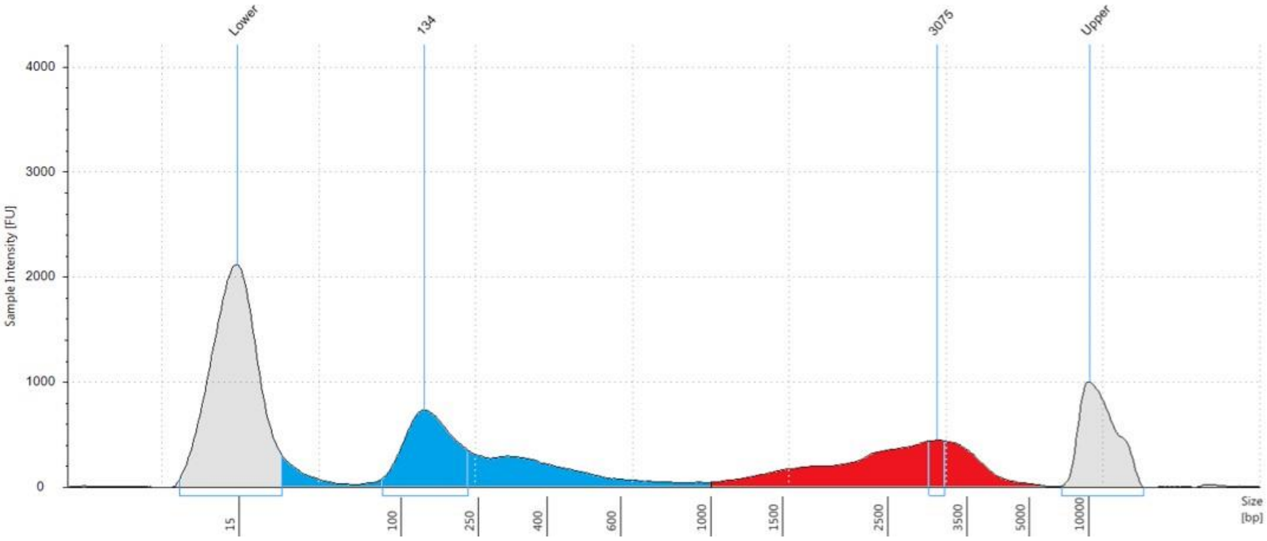

Supplementary Figure S1. Peak area analysis of cfDNA integrity. Example of TapeStation 4200 electopherogram from typical cfDNA sample. Fragments smaller than 1,000 bp were considered to be apoptotic fragments (blue) and larger fragments were considered as necrotic fragments (red). Integrity score (cfDI) was calculated by dividing the peak area of necrotic fragments by the peak area of apoptotic fragments.

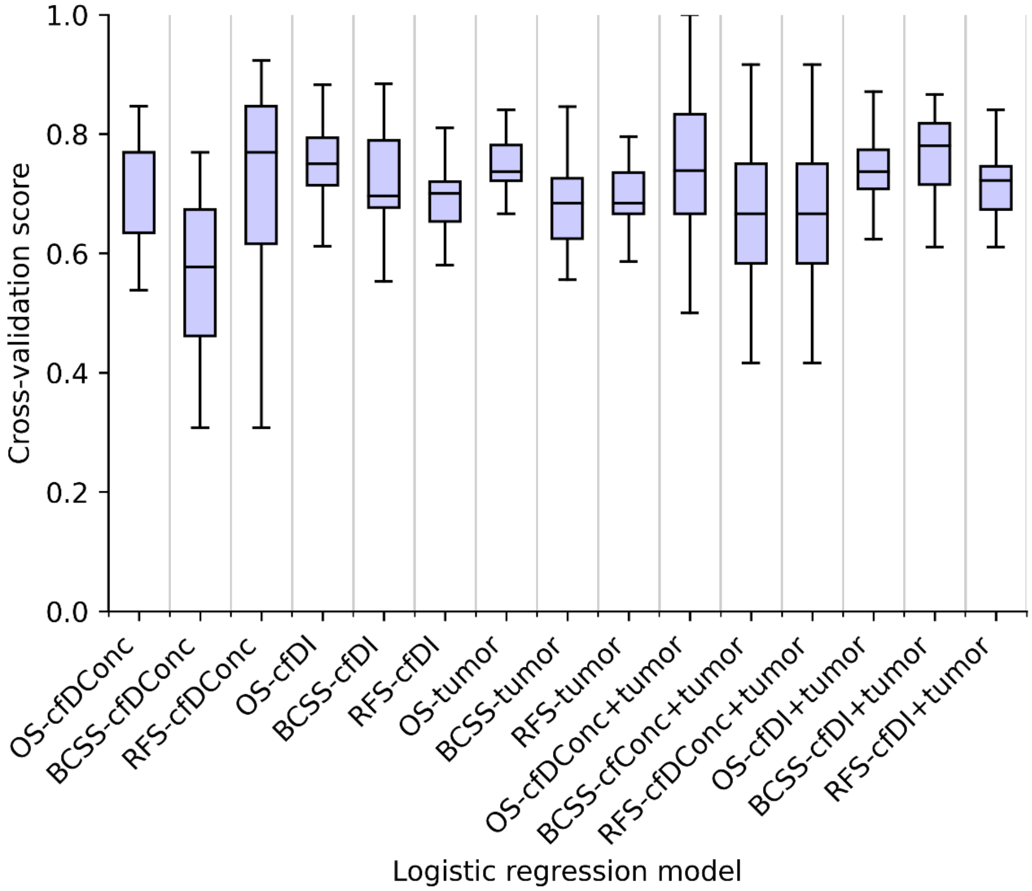

Supplementary Figure S2. Logistic regression models were validated with k-fold cross-validation (k=10, 3 repeats). Validation confirms that univariate models with cfDConc as an only predictor suffer from varying accuracy while models with cfDI provide generally more robust results. Similarly, multivariate logistic regression models with cfDConc as an included predictor show varying accuracy while models with tumor features and cfDI provide more stable results.

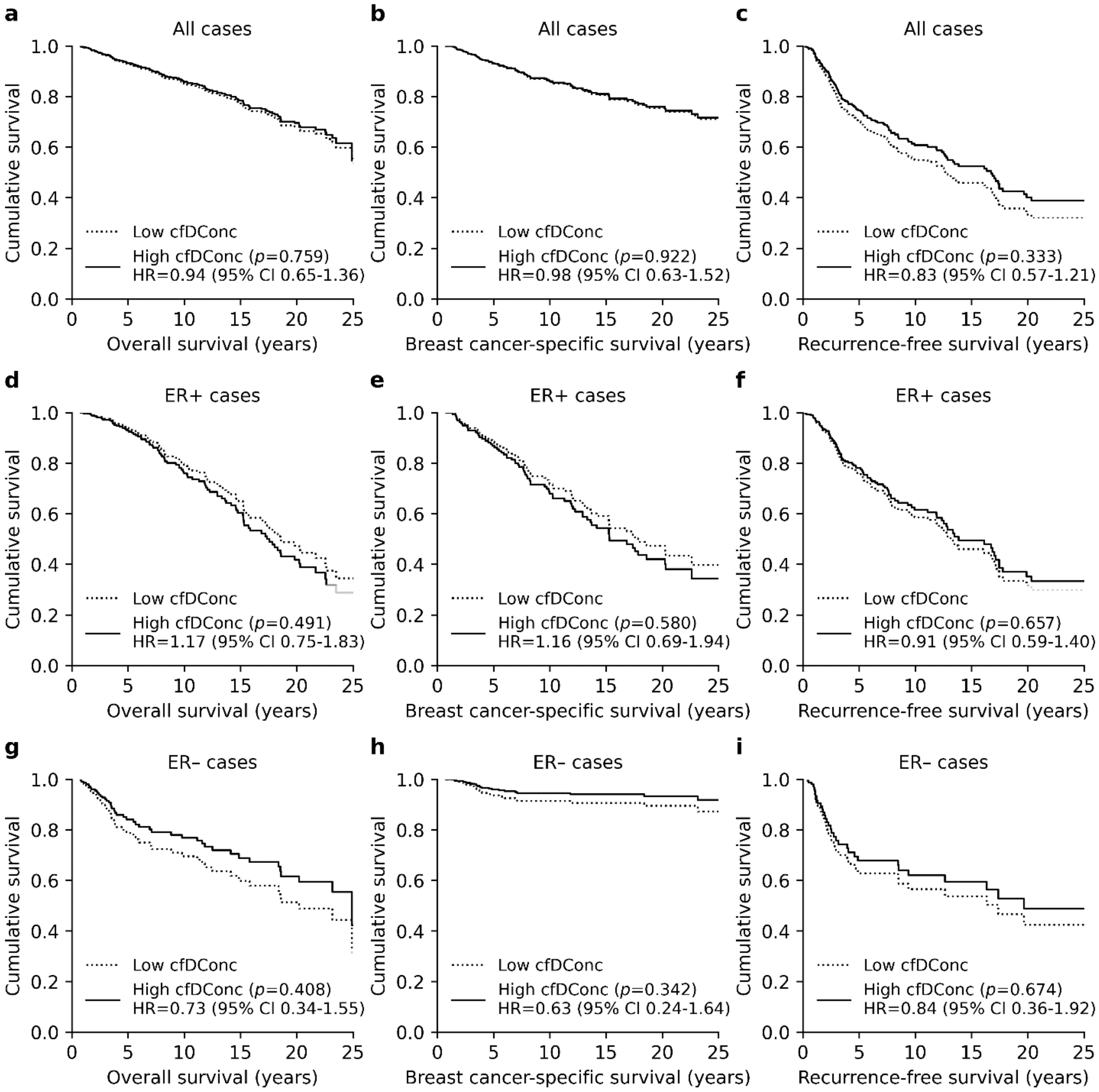

Supplementary Figure S3. Cox regression analysis plots of high cfDConc and BC survival. Multivariate survival analysis did not reveal signi_cant associations between high cfDConc and BC survival. Observed survival was mainly explained by traditional tumor features such as tumor stage and lymph nodal status. Grey lines represent the survival function of high cfDConc group in areas where the plot overlaps with the legend.

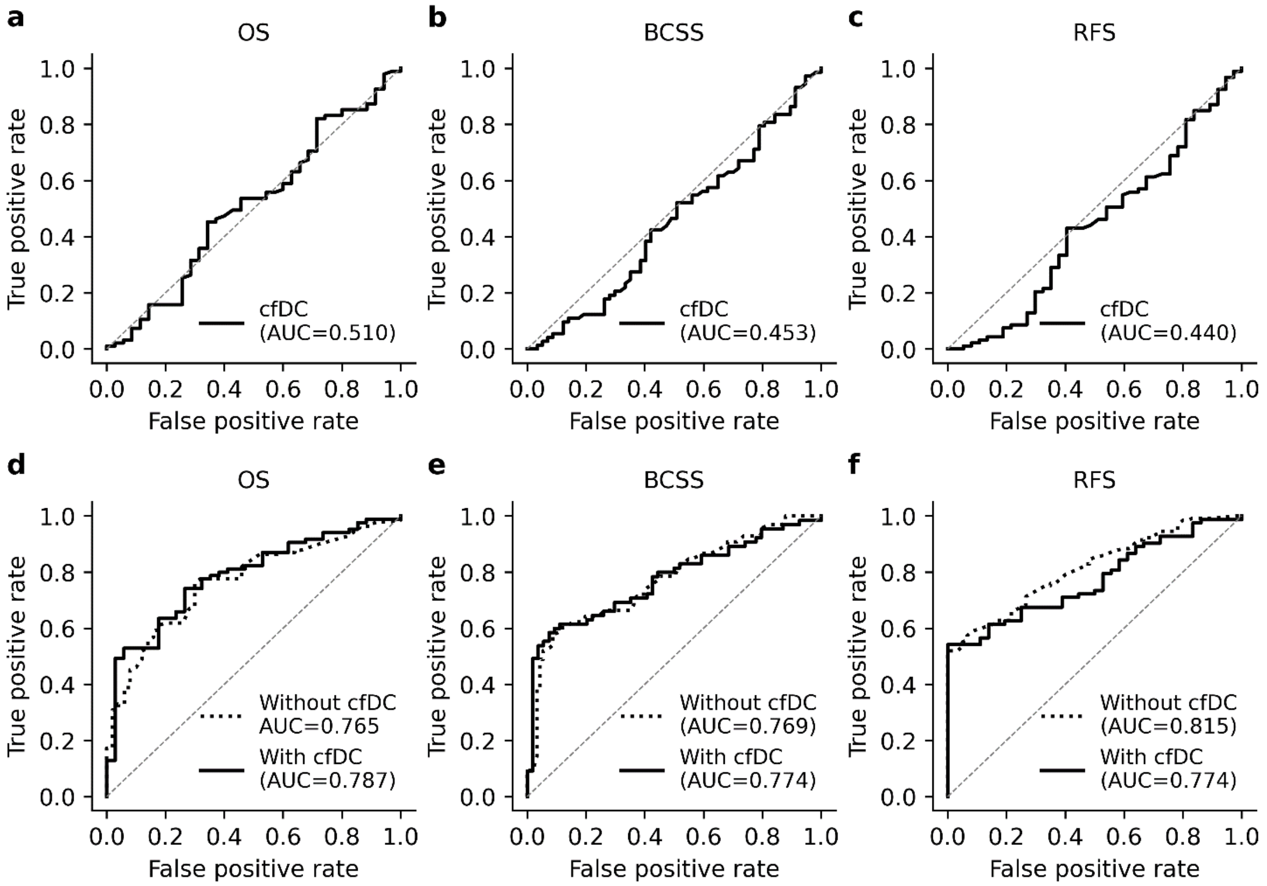

Supplementary Figure S4. Performance of univariate and multivariate logistic regression models with cfDConc. The top row (**a-c**) represents the ROC curves derived from the univariate logistic regression model with cfDConc as a predictor. Univariate models did not significantly differ from random classifier. The bottom row (**d-f**) illustrates the performance of the multivariate logistic regression model with and without cfDConc as a predictor. Curves illustrate that inclusion of cfDConc in the model does not significantly improve the model performance.
